# Forecasting Tuberculosis Incidence in Somalia: A Comparative Analysis of Single and Hybrid Time‐Series Models

**DOI:** 10.1002/hsr2.71923

**Published:** 2026-02-25

**Authors:** Hana Mahdi Dahir, Ayan Husein Korse, Farduus Ibrahim Mohamed, Saralees Nadarajah, Mohamed Said Hassan

**Affiliations:** ^1^ School of Postgraduate Studies and Research Amoud University Amoud Valley Borama Somalia; ^2^ School of Postgraduate Studies and Research University of Hargeisa Hargeisa Somalia; ^3^ Department of Mathematics University of Manchester Manchester UK; ^4^ College of Health Sciences, School of Medicine and Surgery Amoud University Borama Somalia

**Keywords:** forecasting, hybrid models, Somalia, time‐series models, tuberculosis incidence

## Abstract

**Background:**

Tuberculosis (TB) remains a significant public health challenge, necessitating accurate forecasting methodologies to support effective control and prevention strategies. This paper explores the application and comparative performance of single and hybrid time‐series models for forecasting TB incidence trends specifically in Somalia.

**Methods:**

Annual TB incidence data from 2000 to 2022 were sourced from the World Bank to train and evaluate a comprehensive suite of 14 time‐series models. This included five single models—ARIMA, ETS, TBATS, Theta, and NNAR—and nine hybrid model combinations (e.g., ARIMA‐ETS, ARIMA‐TBATS, ARIMA‐ETS‐TBATS). Model performance was assessed using Theil's *U* statistic, Mean Absolute Percentage Error (MAPE), Symmetric Mean Absolute Percentage Error (SMAPE), and Root Mean Square Error (RMSE).

**Results:**

Among the single time‐series models, the TBATS model demonstrated the best fit. However, the comparative analysis revealed that the hybrid ARIMA‐ETS‐TBATS model outperformed other hybrid configurations. The study highlights that hybrid modeling offers enhanced forecasting accuracy compared to single models.

**Conclusion:**

The resulting forecasts provide valuable insights into future TB incidence trends in Somalia. These findings underscore the importance of hybrid modeling in generating accurate data to aid informed public health decision‐making and the development of targeted intervention strategies for TB control.

## 1 Introduction

Tuberculosis (TB) is a chronic infectious disease caused by the bacillus *Mycobacterium tuberculosis*, primarily affecting the lungs, and is recognized as one of the top 10 causes of death worldwide [1, 2, 3]. Tuberculosis remains a major global public health problem [4, 5]. ranking as the second leading infectious disease killer in 2022, with approximately 1.267 million deaths. The rise of multidrug‐resistant TB (MDR‐TB) is a critical concern, with an estimated 410,000 cases reported. Although TB incidence has decreased by 23% from 2015 to 2022, this progress falls short of the End TB Strategy's goal of a 50% reduction by 2025. Notably, around 44 million lives have been saved through TB diagnosis and treatment since 2010. Ending the TB epidemic by 2030 is a key target within the Sustainable Development Goals, highlighting the need for continued efforts in prevention and control. According to the global tuberculosis report in 2023, the total number of new tuberculosis (TB) cases globally was approximately 10.8 million, slightly increasing from 10.7 million in 2022. That is significantly higher than the 10.4 million reported in 2021 and 10.1 million in 2020 [6].

In Africa, TB poses a particularly severe public health challenge, accounting for 2.5 million new cases in 2022, which represents a quarter of the global total. An estimated 424,000 people died from TB in the African region, contributing to over 33% of TB‐related deaths worldwide. The impact of TB is especially pronounced among children, with approximately 1.3 million children falling ill and 250,000 dying from the disease, including those with HIV‐associated TB. TB is also a leading cause of death among HIV‐positive individuals, accounting for 26% of HIV‐related deaths in 2022. Despite a decrease in TB incidence of 8.7% from 2015 to 2022, this reduction is not enough to achieve the End TB Strategy's targets. However, significant progress has been made, with an estimated 10 million lives saved in Africa through TB diagnosis and treatment between 2010 and 2022. The urgent need for continued efforts in TB prevention and control across the continent remains critical [6].

The World Health Organization (WHO) recognizes tuberculosis (TB) as a critical public health issue, especially in low‐ and middle‐income countries where incidence rates are high due to factors like poverty, inadequate healthcare, and co‐morbidities such as HIV/AIDS. Despite global efforts, including the WHO's End TB Strategy aiming for a 90% reduction in new cases by 2035 compared to 2015, challenges persist in controlling the disease [3]. In 2019, Colombia reported a tuberculosis incidence rate of 20 new cases for every 100,000 inhabitants, totaling 14,684 new cases for the year. To help control the disease, innovative approaches such as time series forecasting (TSF) have been studied to provide additional knowledge about the behavior of TB and support public health strategies [7]. With Indonesia ranking second globally for the highest tuberculosis (TB) burden, the country is actively seeking methods to control the disease's spread. Time series forecasting provides a valuable tool for these efforts, allowing public health officials to predict future incidence rates and better prepare intervention strategies [8].

Accurately predicting tuberculosis incidence remains a significant challenge for public health management in China. This study developed a machine learning optimization algorithm to forecast TB trends in Anhui Province, aiming to improve prediction accuracy by using multi‐source data from 2013 to 2023 [9]. In Brazil, where tuberculosis incidence has been increasing since 2015, time series analysis is used to forecast future trends for public health planning. Projections based on these models estimate that Brazil could face 124,245 new cases in 2030, a figure that underscores the challenge of meeting global control targets [10]. Despite a general downward trend in global tuberculosis (TB) incidence from 2000 to 2021, a concerning 3.9% increase occurred between 2020 and 2021. To assess future progress, this global study used the Autoregressive Integrated Moving Average (ARIMA) model to forecast TB incidence, concluding that the World Health Organization's goal of ending the pandemic by 2030 is unlikely to be achieved at the current rate of decline [11].

In Somalia TB is a significant public health issue, with notable changes in incidence rates over the years. In 2018, the TB incidence was recorded at 258 cases per 100,000 population. However, slightly increased to 259 cases per 100,000 in 2020, indicating some fluctuations in the trend. By 2023, the TB incidence had decreased to 246 cases per 100,000 population, reflecting a 14% reduction over the past 14 years [12]. The ARIMA (Autoregressive Integrated Moving Average) model is a traditional mathematical forecasting method used for infectious disease prediction, recognized for its effectiveness in capturing trends and seasonality in time series data [13, 14, 15]. A recent review of studies published between 2012 and 2023 indicates that ARIMA, SARIMA, ETS, GRNN, BPNN, NARNN, NNAR, and RNN models are frequently employed in the prediction of tuberculosis incidence rates [16]. The ARIMA (Auto‐Regressive Integrated Moving Average) model, particularly its seasonal variant SARIMA, has become a valuable tool for forecasting tuberculosis (TB) incidence. By analyzing historical TB data, ARIMA models effectively capture trends, seasonality, and randomness, allowing for accurate predictions of future cases [17].

This study aims to apply the ARIMA, Theta, NNAR, TBATS, and ETS models, alongside hybrid approaches, to forecast tuberculosis incidence in Somalia, providing critical insights into trends and fluctuations in TB cases. By analyzing historical data, the research will support public health officials and policymakers in developing targeted interventions to combat TB effectively. Ultimately, this study contributes to the achievement of Sustainable Development Goal 3 (SDG 3), which seeks to ensure healthy lives and promote well‐being for all, by enhancing TB control efforts in Somalia.

### 1.1 Novelty of the Paper

Despite the critical public health burden of tuberculosis (TB) in the Horn of Africa, a significant gap exists in the literature regarding country‐specific predictive modeling. Current research on TB in Somalia is predominantly retrospective, limited to descriptive epidemiology, annual surveillance summaries, or broad cross‐country aggregations that fail to capture localized epidemiological dynamics. To date, there is no published evidence of comparative time‐series forecasting applied exclusively to Somalia's health data. Consequently, national health authorities lack rigorous, forward‐looking estimates necessary for strategic resource allocation and the precise monitoring of World Health Organization (WHO) reduction targets. Furthermore, there is a methodological deficit in the existing literature regarding the application of advanced forecasting techniques within fragile, data‐scarce health systems; few studies address how to derive robust predictions from small sample sizes in conflict‐affected regions.

To address these critical voids, this study establishes its novelty by conducting the first comprehensive predictive analysis of annual TB incidence specifically for Somalia. We fill the identified methodological gap by rigorously evaluating fourteen forecasting approaches, including classical statistical models (ARIMA, ETS, TBATS, Theta), machine‐learning autoregressive architecture (NNAR), and multiple hybrid ensembles. While previous studies have not utilized historical annual TB incidence data to model medium‐term trends in this setting, this work demonstrates the feasibility of combining statistical models to improve forecast robustness under constrained data availability. A central innovation is the development and validation of a hybrid ARIMA–ETS–TBATS ensemble, which integrates linear trend dynamics, exponential smoothing structure, and complex seasonal components. This hybrid approach consistently outperformed individual models across multiple train–test schemes and was statistically supported using Model Confidence Set testing. Accordingly, the present study offers the first localized, time‐series–based TB forecast for Somalia, while providing a transferable methodological framework for other low‐resource settings that experience similar limited epidemiological data, inconsistent surveillance, and structural reporting gaps.

## 2 Materials and Methods

### 2.1 Study Area and Source of Data

The study utilized a time series design, which is a form of longitudinal study. Time series analysis involves various methods that decompose time series data into identifiable components, enabling the recognition of trends and the generation of forecasts. This study focuses on foundational models such as ARIMA, Theta, NNAR, TBATS, and ETS to analyze tuberculosis incidence in Somalia from 2000 to 2023, exploring both individual model performance and potential improvements through hybrid approaches. The data, sourced from the World Bank database, includes annual tuberculosis incidence rates measured per 100,000 population, providing a robust foundation for forecasting future trends in TB incidence. To evaluate model performance, the time‐series data were chronologically partitioned into a training set (2000–2017) and a validation set (2018–2022). This hold‐out method provides a single, out‐of‐sample test to assess how well the models generalize to unseen data. While more advanced techniques like rolling‐window validation were not utilized, this strict separation ensures a fair assessment of robustness. Consequently, all reported performance metrics are based exclusively on the validation set to reflect true forecasting accuracy.

### 2.2 Stationarity Tests

Stationarity tests are fundamental in time series analysis, as they determine whether a data set's statistical properties remain constant over time. The Augmented Dickey‐Fuller (ADF) test and the Phillips‐Perron (PP) test are two widely used methods for assessing stationarity. The ADF test evaluates the presence of a unit root, where the null hypothesis suggests that the series is non‐stationary. If the test statistic is more negative than the critical value, the null hypothesis can be rejected, indicating that the series is stationary. Similarly, the PP test also checks for unit roots but incorporates drift and trend in the data.

The ADF test is a widely used statistical test designed to determine whether a time series is stationary or has a unit root, indicating non‐stationarity.

The ADF test extends the basic Dickey‐Fuller test by including lagged differences of the time series to account for higher‐order serial correlation. The null hypothesis of the ADF test posits that the time series has a unit root (i.e., it is non‐stationary). In contrast, the alternative hypothesis suggests that the time series is stationary. The ADF test can be applied to the equation of the form: The ADF test statistic is calculated as follows:

$$\mathrm{ADF}=\frac{\mathrm{Coefficient}\;\mathrm{Estimate}-1}{\mathrm{Standard}\;\mathrm{Error}}$$

The Phillips‐Perron (PP) test is another statistical test for determining the presence of a unit root in a univariate time series, which helps assess whether the series is stationary or non‐stationary. Unlike the Augmented Dickey‐Fuller (ADF) test, which uses lagged values to correct for serial correlation, the PP test employs a nonparametric correction method to handle autocorrelation and heteroscedasticity in the error terms, making it more flexible in some scenarios. The null hypothesis of the PP test posits that the time series has a unit root (indicating non‐stationarity), while the alternative hypothesis asserts that the series is stationary. The PP test can be expressed through the regression equation: The PP test statistic is calculated as follows:

$$\mathrm{PPT}=\frac{\mathrm{Coefficient}\;\mathrm{Estimate}-1}{\mathrm{Standard}\;\mathrm{Error}}$$

Based on the ADF and PP stationarity tests, the original and first‐differenced TB incidence series remained non‐stationary (*p* > 0.05), whereas second differencing resulted in statistical stationarity (*p* < 0.05). Therefore, ARIMA models in this study were estimated using a differencing order of *d* = 2. The use of second differencing was empirically justified to remove long‐term trend components without introducing noise.

### 2.3 Time Series Models

#### 2.3.1 Single Models

##### 2.3.1.1 The ARIMA Model

ARIMA model was proposed by Box and Jenkins in the early 1970s, so it is also called Box‐Jenkins model and Box‐Jenkins method [18]. It is the most commonly used prediction technique in the evaluation and monitoring of epidemiological surveillance. ARIMA is an extension of the autoregressive moving average model (ARMA), combining difference operation and the ARMA model [19, 20].

The general form of the ARIMA models is written as follows: ARIMA (*p*, *d*, *q*) × (*P*, *D*, *Q*)*s*, where *p* is the number of parameters in the autoregressive (AR) model, d the differencing degree, *q* the number of parameters in the MA model, *P* the number of parameters in AR seasonal model, *D* the seasonal differencing degree, *Q* the number of parameters in MA seasonal model, and *s* the period of seasonality. Because there was a strong seasonality trend in this study, we constructed a seasonal ARIMA (*p*, *d*, *q*) × (*P*, *D*, *Q*)*s* model. Before fitting the ARIMA model, an appropriate differencing of the series is usually performed to make the series stationary. If the series is not stationary, differencing can be used to transform it into a stationary series [21].

where p represents the number of lag observations included in the model, *d* is the degree of differencing needed to achieve stationarity, and *q* is the size of the moving average window [22]. An ARMA model, characterized by the parameters *p* and *q*, is constructed using historical values from the series {*Yt*} along with random disturbances {∈*t*}. This relationship is represented by the following equations.

y_t=∑_(i=1)^p∅_iy_(t−i)+∑_(j=1)^qΘ_jε_(t−j)+ε_t

To separate the influence of previous observations from the random errors, equation was modified by positioning the autoregressive components on one side and the moving average components on the other side. This results in the following equation:

y_t=∑_(i=1)^pθ_iy_(t−i)+∑_(j=1)^qθ_jε_(t−j)+ε_t

Where:

- *p* = number of autoregressive (AR) terms
- *d* = number of differencing steps applied to achieve stationarity
- *q* = number of moving average (MA) terms
- *B* = backshift operator such that Byt = yt−1
- *ϕ*(*B*) and *θ*(*B*) represent the AR and MA polynomials, respectively
- *εt* = white‐noise error term

In this study, the optimal ARIMA model was selected using the auto.arima() function in R, which automatically evaluates multiple model configurations and chooses the best‐fitting parameters based on the Akaike Information Criterion corrected for small samples (AICc). Differencing was applied only as required to achieve stationarity, and second differencing (*d* = 2*d* = 2*d* = 2) was selected because it minimized AICc and ensured a stable residual structure. All model estimation procedures used default R settings unless stated otherwise, ensuring full reproducibility.

##### 2.3.1.2 TBATS

The TBATS model, which stands for Trigonometric, Box‐Cox transformation, ARMA errors, Trend, and Seasonal, is a sophisticated forecasting technique specifically designed to handle complex seasonal patterns in time series data. It incorporates several advanced statistical components, including trigonometric functions to accommodate multiple seasonal cycles, Box‐Cox transformations to manage non‐linear relationships, and autoregressive moving average (ARMA) error structures to capture temporal dependencies. Unlike traditional models such as SARIMA, which typically require integer‐seasonal parameters and assume linear relationships, TBATS is flexible enough to handle non‐integer seasonality and various types of non‐linearity, making it particularly useful for forecasting in domains such as epidemiology, where data often exhibit intricate seasonal trends and patterns. Its robustness in providing more accurate forecasts has made it an increasingly popular choice for analyzing and predicting the incidence of infectious diseases [23, 24],

Y^(ω)=I_(t−1)+∑_(t=1)^TS_(t−m_1)^(i)+D_t

Where Yt(*ω*) is the Box–Cox–transformed observation at time It−1 represents the local level component from the previous period, St−mi(i) denotes the trigonometric seasonal component for the ith seasonal cycle shifted by mi, *T* is the number of seasonal cycles modeled, and Dt captures ARMA‐based short‐term error dynamics.

##### 2.3.1.3 ETS

ETS (Error, Trend, Seasonality) models provide a statistical framework for exponential smoothing, decomposing time series data into level, trend, and seasonal components. This allows for systematic parameter estimation, the construction of prediction intervals, and the selection of different exponential smoothing variations based on the specific characteristics of the data. The ETS framework offers a robust and widely used approach to forecasting by capturing underlying patterns in time series [25].

y_t=w(x_(t−1))+r(x_(t−1))ε_t and x_t=f(x_(t−1))+g(x_(t−1))ε_t

In this formulation, yt denotes the forecast at time *t*, xt−1 represents the previous smoothed level of the series, *ω* is the smoothing parameter controlling the influence of the level component, *ρ* determines the contribution of the error term, and *εt* captures random fluctuations at time *t*.

##### 2.3.1.4 NNAR

NNAR (Neural Network Auto Regression) models leverage the power of neural networks to forecast time series data by capturing complex, non‐linear relationships that traditional linear models often miss. Using past values of the time series as inputs, NNAR models learn intricate patterns and dependencies, making them particularly effective for forecasting data with irregular fluctuations or non‐linear trends. This approach offers a flexible and adaptable alternative to conventional time series methods [26].

The NNAR model is a time‐series forecasting approach that employs neural networks to identify patterns and relationships within the data. It integrates autoregressive elements with the non‐linear features of neural networks to enhance forecasting precision. The specific formula for the NNAR model varies based on its architecture.

y_t=ω_0+∑_(k=1)^Qα_k(θ_k+∑_(i=1)^Pφ_iy_(t−i))+e_t

In this expression, yt represents the forecast at time *t*, *ω*_0_ is the bias term, *P* denotes the number of autoregressive lags used as neural network inputs, *ϕi* are the autoregressive weights applied to past observations, *Q* is the number of hidden neurons, *αk* and *θk* are the neural network activation weights and biases at the hidden layer, and *et* captures random error at time *t*.

##### 2.3.1.5 Theta

The Theta model is a simple yet effective time series forecasting method that decomposes the original series into two “Theta lines.” These lines are constructed using a modified version of the original data, one with an adjusted local level and the other with an adjusted local trend. By forecasting these Theta lines separately and then combining the forecasts, the model captures both level and trend information, often providing surprisingly accurate short‐term predictions with minimal complexity [27].

^Yt=12(^Yt0=0+^Yt0=2)

In this equation, y^t (*θ* = 0) represents the forecast derived from the damped trend component of the series, while y^t (*θ* = 2) represents the forecast from the adjusted level component; the final Theta forecast y^t is obtained by averaging both components, allowing the model to capture both smooth trend and curvature behavior in the underlying time series.

#### 2.3.2 Hybrid Models

##### 2.3.2.1 Hybrid Modelling Approaches

In this study, a hybrid modeling approach was employed to improve the accuracy of forecasts for Tuberculosis (TB) incidence in Somalia. The core rationale is that relying on a single model whether linear like ARIMA or non‐linear like NNAR—may not fully capture the complex patterns inherent in epidemiological time series data. By integrating different models, we can harness their complementary strengths; for example, ARIMA effectively models linear autoregressive patterns, while NNAR is adept at identifying intricate, non‐linear relationships. The implementation involved constructing a range of hybrid models, including two‐ and three‐component configurations such as ARIMA‐ETS, ARIMA‐TBATS, ARIMA‐NNAR, and the more complex ARIMA‐ETS‐TBATS. These models were developed using the forecastHybrid package in R, which constructs hybrid forecasts using an *ensemble forecasting* methodology. The specific hybridization process involved two steps: first, each individual model component (e.g., ARIMA, ETS) was fitted independently to the training data to generate its own forecast. Second, these individual forecasts were combined into a single, final hybrid forecast. As specified in our R code by the weights = “equal” parameter within the hybridModel() function, the combination method used was *equal‐weighted averaging*, meaning the final forecast is the simple arithmetic mean of the predictions from each constituent model. It is important to distinguish this ensemble approach from other hybrid techniques, such as sequential residual modeling, which were not employed. A key feature of this methodology was the automatic selection of hyperparameters for each model such as ARIMA's (*p*, *d*, *q*) orders—based on optimized information criteria during the fitting process. The primary goal of this comprehensive approach was to generate more robust and precise forecasts by synergistically modeling the data's linear, non‐linear, and structural components, a goal successfully achieved by the top‐performing ARIMA‐ETS‐TBATS model. Unlike sequential hybridization approaches, where residuals from a primary model (e.g., ARIMA) are modeled using a secondary method, this study employed a parallel ensemble mechanism in which ARIMA, ETS, TBATS, and other component models were independently trained, and their forecasts combined using equal weights. This choice was made to avoid instability caused by residual‐modeling when working with a small annual data set.

#### 2.3.3 Performance Metrics

##### 2.3.3.1 SMAPE

The Symmetric Mean Absolute Percentage Error (SMAPE) is a crucial metric used to assess the accuracy of forecasting models. This metric evaluates the percentage difference between forecasted values and actual observed values, while also considering the magnitude of the actual values. SMAPE effectively mitigates the impact of significant discrepancies by normalizing the errors concerning the exact values, thus providing a more balanced view of forecasting accuracy.

SMAPE=(1/n)∑_(i=1)^n[(|F_i−A_i|)/((|F_i|+|A_i|)/2)] × 100

In this context, Fi denotes the forecasted value at a specific time *i*, while Ai represents the actual value at that same time. SMAPE quantifies the absolute percentage difference between each observation's predicted and actual values. This difference is then normalized by taking the average of the absolute forecasted and actual values and expressed as a percentage. By averaging these individual percentage differences, one arrives at the overall SMAPE score, which reflects the average forecasting error relative to the actual values as a percentage.

##### 2.3.3.2 Theil's *U* Statistics

Theil's *U* statistic is a commonly utilized metric for assessing forecasting accuracy and comparing different models within econometrics. It offers an in‐depth evaluation of how various forecasting models perform relative to one another by considering the forecasted and actual observed values. The formula used to calculate Theil's *U* statistic is as follows:

U=√[((1/n)∑_(t=1)^n((A_t−F_t)/A_t)²)/((1/n)∑_(t=1)^n((A_t−A_(t−1))/A_(t−1))²)]

The formula's numerator calculates the root mean square forecast error, which assesses how accurately the forecasted values align with the observed values. In contrast, the denominator reflects a naive forecast's root mean square error, where the average of the observed values serves as the forecast for every time period. Dividing these two components normalizes the forecast error against the error of the naive forecast, enabling a more meaningful comparison among various models or forecasting methods.

##### 2.3.3.3 MAPE

MAPE assesses the average percentage deviation between predicted and actual values. It is particularly valuable for comparing forecasting accuracy across different time series scales. The formula for calculating MAPE is

MAPE=(1/n)∑_(t=1)^n[(|A_t−F_t|)/|A_t|] × 100

In this context, (yt) refers to the actual value at time (*t*), (ˆ yt) indicates the predicted value at time (*t*), (^−^y) signifies the average of the observed values, and (*n*) represents the overall count of observations.

##### 2.3.3.4 RMSE

Root Mean Square Error (RMSE) is a widely used metric for evaluating the accuracy of a predictive model by measuring the average magnitude of the errors between predicted and observed values. It is calculated by taking the square root of the average of the squared differences between predicted values (*ŷ*) and actual values (*y*). RMSE provides a sensitive measure of how well the model performs; lower RMSE values indicate better model accuracy, while higher values suggest more significant error in predictions. This metric is handy in regression analysis and forecasting, as it emphasizes more significant errors due to the squaring process, making it effective for identifying substantial deviations.

RMSE=√[(1/n)∑_(i=1)^n(ŷ_i−y_i)²]

##### 2.3.3.5 Differencing

Differencing is an essential technique in time series analysis used to stabilize the mean by removing trends and seasonality, making the data more stationary. In this study, differencing is applied to *Tuberculosis Incidence in* from Somalia to address non‐stationarity, which can impact the accuracy of the Auto‐Regressive Integrated Moving Average (ARIMA) model. By calculating the differences between consecutive observations, we enhance the model's predictive capabilities and ensure that the assumptions of the ARIMA model are met, leading to more reliable forecasts of female unemployment trends from 2000 to 2023.

###### 2.3.3.5.1 Diebold‐Mariano (DM) Test

To determine if the difference in predictive accuracy between two competing models is statistically significant, we utilized the test proposed by Diebold and Mariano [28]. Given two forecast series with errors e1t*e*1*t* and e2t*e*2*t*, we defined the loss differential series dt*dt* using a squared‐error loss function (L(et)=et2*L*(*et*)=*et*2), such that dt=e1t2−e2t2*dt*=*e*1*t*2−*e*2*t*2 The null hypothesis of equal predictive accuracy is H0:E[dt]=0*H*0:*E*[*dt*]=0. The test statistic is defined as:

DM=d−V^(d−)/T

where *d* ^−^ is the sample mean of the loss differential, *T* is the number of forecasts, and *V*^(*d*ˉ) is the consistent estimate of the asymptotic variance of d^−^ *d* ^−^. A statistically significant negative DM statistic indicates that Model 1 has significantly lower forecast errors than Model 2.

###### 2.3.3.5.2 Model Confidence Set (MCS)

To address the limitations of pairwise comparisons when evaluating multiple models, we applied the Model Confidence Set (MCS) procedure introduced by [29]. The MCS procedure identifies a subset of models (the Superior Set of Models, SSM) that are statistically indistinguishable from the “best” model at a given confidence level (1 − *α*1 − *α*). We employed the Semi‐Quadratic loss function for the MCS evaluation. The procedure involves a sequence of equivalence tests (δM) based on the null hypothesis that all models in the candidate set M*M* have equal expected loss. If H0 is rejected, the worst‐performing model is eliminated using an elimination rule (eM, and the test is repeated on the reduced set. We used the Block Bootstrap method (*R* = 5000 replications) to account for time‐series dependency when deriving the distribution of the test statistic (*T*max).

### 2.4 Data Preprocessing

This study utilizes annual data on the incidence of Tuberculosis in Somalia, sourced from the World Bank's World Development Indicators. The dataset comprises 23 observations spanning the years 2000 to 2022. Prior to model fitting, the dataset underwent several preprocessing steps to ensure its suitability for time series analysis. This included a thorough examination for missing values and outliers, and the data set was found to be complete and continuous, requiring no imputation. The most critical preprocessing step involved assessing the stationarity of the time series using the Augmented Dickey‐Fuller (ADF) and Phillips‐Perron (PP) tests. As these tests confirmed the original series was non‐stationary, first‐differencing was applied to stabilize the mean and prepare the data for the modeling process. All data preprocessing, including stationarity testing, differencing, and train–test partitioning, was conducted in R (version 4.X). The analysis made use of the forecast, forecastHybrid, TSstudio, and tseries packages, which ensured standardized implementation of statistical tests and model‐fitting routines. All model estimation and validation procedures were executed within the same software environment to ensure full reproducibility.

### 2.5 Statistical Analysis

All statistical analyses were conducted using R software (version 4.3.1), primarily utilizing the forecast and forecast Hybrid packages. The development of the ARIMA model employed in this study involves four key steps. First, data preparation is conducted using the Augmented Dickey‐Fuller (ADF) test to assess the stationarity of the time series. If the series is found to be non‐stationary, data differencing is applied until stationarity is achieved. The number of differencing operations performed corresponds to the values of *d* and *D* in the model. In this study, the ADF tests on the original data revealed that the series was not stationary (*p* > 0.05). Therefore, we applied one non‐seasonal difference (*d* = 1) and one seasonal difference (*D* = 1) to stabilize the incidence series.

Second, the parameters *p* and *q* were identified. We established fixed values for (*p*, *q*) to create suitable models by analyzing the autocorrelation function (ACF) and partial autocorrelation function (PACF) plots of the stationary series. This step is essential for determining the optimal lag values that enhance the model's forecasting accuracy [2].

## 3 Results

### 3.1 Descriptive Statistics of Data

Table 1 presents the descriptive statistics for tuberculosis incidence in Somalia. The average incidence rate is 27.25, with a standard deviation of 0.43, indicating low variability around the mean. The median value of 27.4 is close to the mean, further confirming the consistency of the data. The minimum and maximum rates are 26.1 and 27.7, respectively, resulting in a range of 1.6. The skewness of −1.5 suggests a leftward skew in the distribution, while the kurtosis value of 1.873 indicates a flatter distribution with fewer extreme values. Overall, these statistics reflect a stable and consistent tuberculosis incidence rate in Somalia during the observed period.

**Table 1:** Descriptive statistics tuberculosis incidence in Somalia.

Mean	SD	Median	Trimmed	MAD	Minimum	Maximum	Range	Skewness	Kurtosis
27.25	0.43	27.4	27.33	0.3	26.1	27.7	1.6	−1.5	1.873

**Figure 1:**
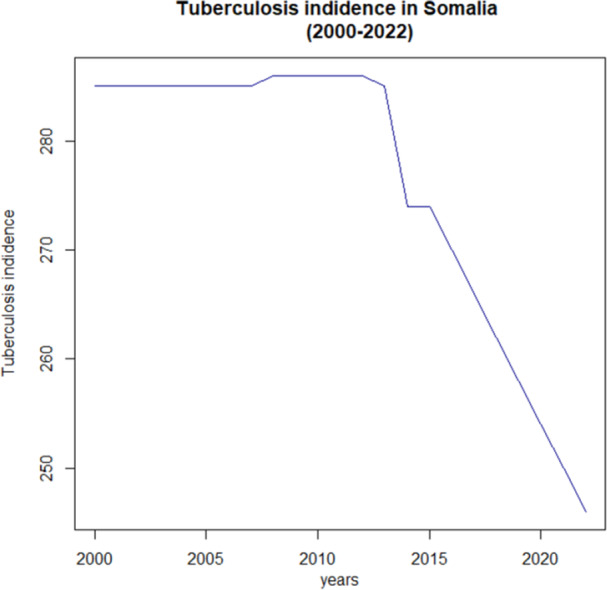
Tuberculosis (TB) incidence in Somalia from 2000 to 2022.

As shown in Figure 1, the trend of Tuberculosis (TB) incidence in Somalia from 2000 to 2022 reveals a non‐linear pattern, indicating that the data is not stationary. The incidence rates exhibit noticeable changes over time rather than maintaining a consistent trend.

**Figure 2:**
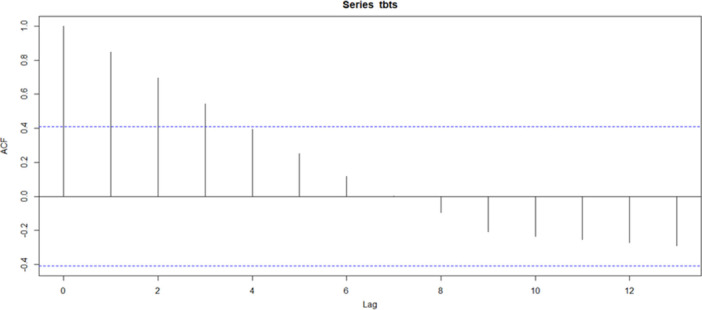
ACF plot of Tuberculosis (TB) incidence trend.

As shown in Figure 2, the autocorrelation function (ACF) plot for the TB incidence data reveals a strong positive autocorrelation at lower lags, particularly between lag 1 and lag 3, indicating a significant relationship between past and current TB incidence values. This characteristic is important for forecasting. As the lag increases, the ACF values gradually decrease toward zero, indicating that the influence of earlier observations diminishes over time. The gradual decline in autocorrelation, with several values exceeding the upper confidence limit, suggests possible non‐stationarity in the series. This highlights the need for differencing or other transformations before applying ARIMA modeling.

**Figure 3:**
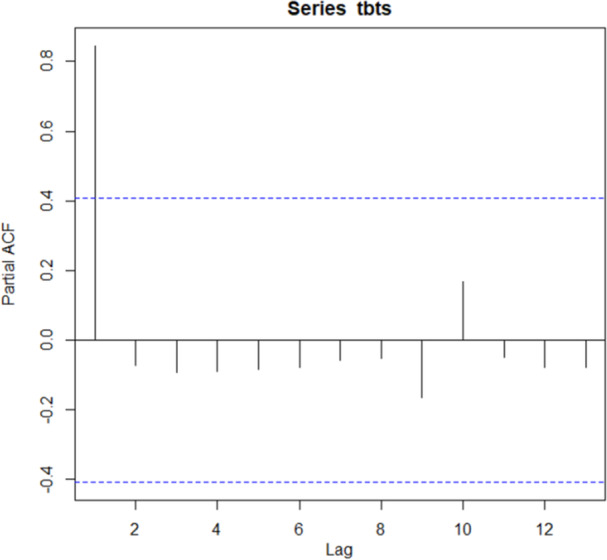
PACF plot of Tuberculosis (TB) incidence trend.

As shown in Figure 3, the Partial Autocorrelation Function (PACF) plot of Tuberculosis (TB) incidence indicates significant partial autocorrelations at lags 1 and 2, with the first lag reaching nearly 0.9. This suggests a strong direct relationship between the current value and its immediate past values. As the lags increase, the PACF values drop and fluctuate around zero, indicating that the influence of earlier values diminishes over time. The horizontal dashed lines represent the confidence intervals, and the lack of significant spikes beyond lag 2 suggests that an autoregressive model of order 2 (AR(2)) may be appropriate for modeling this time series.

Table 2 presents the results of the Augmented Dickey‐Fuller (ADF) and Phillips‐Perron (PP) tests for stationarity. The ADF test for the original and first‐differenced data shows non‐significant *p*‐values (0.9742 and 0.4291, respectively), indicating that the data remains non‐stationary. However, after second differencing, the ADF test becomes significant (*p* = 0.03636), suggesting stationarity. Similarly, the PP test indicates non‐stationarity in the original data (*p* = 0.99) but shows significance at first and second differencing (*p* = 0.01 for both), confirming stationarity after differencing. These results collectively imply that the TB incidence data requires at least second differencing to achieve stationarity for ARIMA modeling.

**Table 2:** Test for stationarity at levels.

Test	Test statistics	*p*‐value
ADF (original data)	0.51015	0.9742
ADF (first differencing data)	2.3761	0.4291
ADF (second differencing data)	3.7909	0.03636
PP (original data)	0.36308	0.99
PP (first differencing data)	25.166	0.01
PP (second differencing data)	28.763	0.01

**Figure 4:**
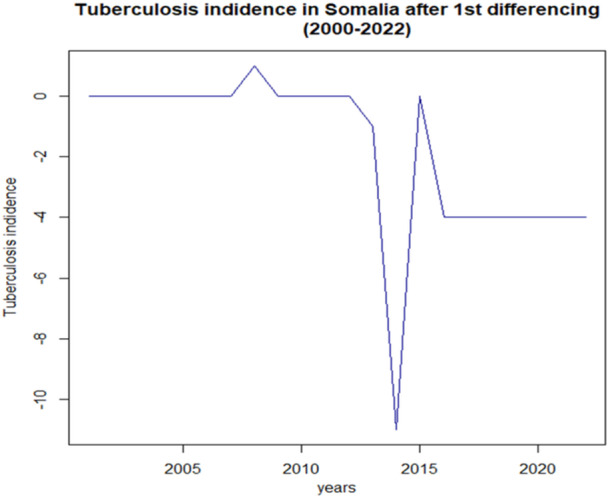
First differencing of tuberculosis incidence in Somalia.

Figure 4 illustrates the first differencing of tuberculosis incidence in Somalia from 2000 to 2022 revealing fluctuations in the data, particularly notable drops around 2005 and 2015. The horizontal axis indicates the years, while the vertical axis shows the change in incidence rates. The sharp decline in 2015 suggests a significant event or intervention impacting tuberculosis rates, while the overall trend appears relatively stable with minor variations, indicating a possible need for further investigation into the factors influencing these changes.

**Figure 5:**
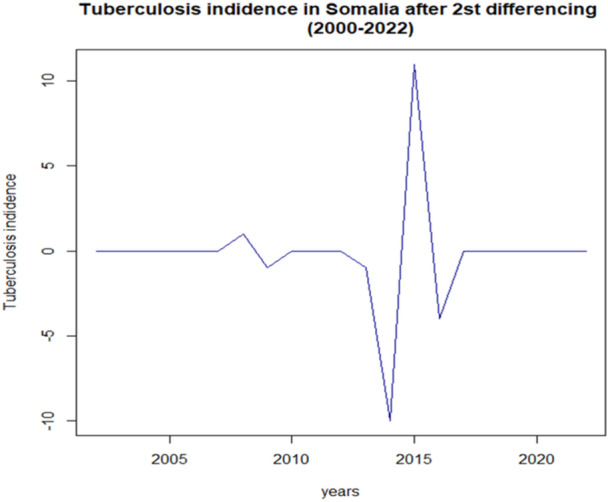
First differencing of tuberculosis incidence in Somalia.

As represented Figure 5, the second differencing of tuberculosis incidence in Somalia from 2000 to 2022 reveals a more stabilized series, with fluctuations indicating changes in incidence rates after accounting for trends and seasonality. The notable spike in 2015 suggests a significant shift or event affecting tuberculosis rates during that period. Overall, the series appears to oscillate around zero, indicating that the underlying trend has been effectively removed, allowing for a clearer analysis of short‐term variations and potential cyclical patterns in the data.

**Figure 6:**
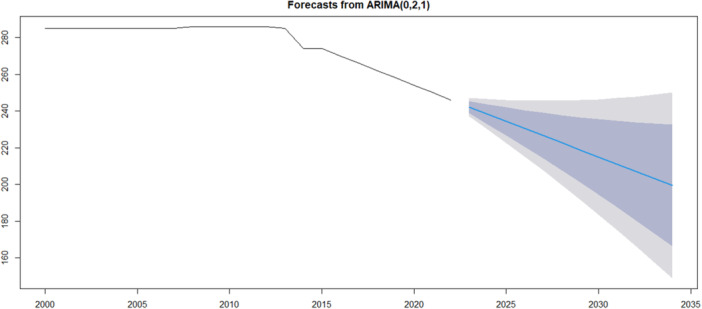
ARIMA model forecast for Tuberculosis incidence in Somalia.

As displaced in Figure 6, the ARIMA (0,2,1) model forecasts for tuberculosis incidence in Somalia predict a gradual decline in cases from 2022 to 2030, with the forecasted values stabilizing around 200. The blue line indicates the estimated future incidence, while the shaded region represents the confidence intervals, reflecting the uncertainty in the forecasts. The projections suggest that while there may be fluctuations, the overall trend points towards a decrease in tuberculosis incidence over the forecast period, potentially influenced by ongoing public health interventions or changes in disease dynamics.

### 3.2 Model Selection

Table 3 indicates that the ARIMA (0,2,1) model was the one with the lowest Akaike Information Criterion (AIC) and the lowest Bayesian Information Criterion (BIC), with values of 103.3040 and 105.3930, respectively. This suggests that the ARIMA (0,2,1) model provides a favorable balance between goodness of fit and model complexity, making it the most appropriate choice for analyzing the data in this study.

**Table 3:** Estimation findings of ARIMA models.

*p*	*D*	*q*	AIC	BIC
1	2	1	104.0507	107.1843
2	2	1	105.7904	109.9685
3	2	0	105.7574	109.9354
0	2	1	103.3040	105.3930

As illustrated in Table 4, the performance metrics for the ARIMA model on the training set indicate a good fit to the data. The model's Mean Error (ME) is 0.1168, suggesting minimal bias in its predictions. The Root Mean Square Error (RMSE) of 0.3135 and Mean Absolute Error (MAE) of 0.1168 indicate that the model maintains low error levels, with the RMSE highlighting the model's ability to minimize larger deviations. The Mean Percentage Error (MPE) and Mean Absolute Percentage Error (MAPE), both at 4.09%, demonstrate that the model's predictions are relatively accurate on a percentage basis. Finally, the Mean Absolute Scaled Error (MASE) of 1.1681 shows that the model's performance is slightly above a baseline prediction model, confirming its reliability for forecasting. Overall, these metrics suggest that the ARIMA model is well‐suited for analyzing and predicting TB incidence trends.

**Table 4:** Error metrics for trail forecast.

	ME	RMSE	MAE	MPE	MAPE	MASE
Training set	0.1168182	0.3135174	0.1168182	0.0408773	0.0408773	1.168182

As detailed in Table 5, the forecast performance of the single time series models reveals varying levels of accuracy, with TBATS demonstrating the best overall performance across several metrics. It achieved the lowest Theil's *U* (2.79), indicating a strong predictive capability relative to the actual values, and the lowest Mean Absolute Percentage Error (MAPE) of 3.46%, suggesting minimal average percentage error in its forecasts. Additionally, TBATS had the lowest Root Mean Square Error (RMSE) at 10.58, reflecting its effectiveness in minimizing the magnitude of forecast errors. In contrast, the ARIMA model exhibited the highest Theil's *U* (7.06) and RMSE (26.25), indicating poorer performance in comparison to the other models. Overall, TBATS stands out as the most reliable model for forecasting in this analysis, while ARIMA may require further refinement or alternative approaches to enhance its predictive accuracy.

**Table 5:** Forecast performance of the single time series models including, ARIMA, TBATS, NNAR, Theta, ETS.

Model	Theil'sU	MAPE	SMAPE	RMSE
ARIMA	7.060884	9.5978543	0.09093386	26.25186
ETS	4.58129	5.5162779	0.05304338	16.73412
NNAR	6.042329	7.939412	0.07571176	22.28412
Theta	4.429601	5.33097	0.05132564	16.17844
TBATS	2.789202	3.4597097	0.03390387	10.584486

As shown in Table 6, the forecast performance of the hybrid time series models indicates a generally improved accuracy compared to the individual models, with ARIMA‐TBATS demonstrating the best results overall. It achieved a Theil's *U* of 4.80, a MAPE of 5.88%, and an RMSE of 17.53, reflecting its effectiveness in capturing the underlying patterns in the data while minimizing forecast errors. Similarly, the ARIMA‐ETS‐TBATS model also performed well, with a Theil's *U* of 4.72 and the lowest MAPE of 5.76%, indicating strong predictive capabilities. In contrast, the ARIMA‐NNAR model exhibited a notably low Theil's *U* of 0.08, but its higher RMSE of 24.01 suggests it may not be as reliable in practice. Overall, the hybrid models, particularly those incorporating TBATS and ETS, show significant promise in enhancing forecast accuracy, highlighting the benefits of combining different modeling approaches to leverage their strengths.

**Table 6:** Forecast performance of the hybrid time series models including ARIMA‐ ETS, ARIMA‐ NNAR, ARIMA‐ Theta, ARIMA‐ TBATS, ARIMA‐ETS‐ Theta, ARIMA‐ETS‐ NNAR, ARIMA‐ ETS‐ TBATS, ARIMA‐ Theta‐ NNAR, ARIMA‐ TBATS‐ NNAR.

Model	Theil's *U*	MAPE	SMAPE	RMSE
ARIMA‐ETS	5.802318	7.557066	0.07217483	21.36612
ARIMA‐NNAR	0.08243115	8.668133	0.08243115	24.0103
ARIMA‐Theta	5.726241	7.459411	0.07128441	21.08669
ARIMA_TBATS	4.795244	5.880628	0.0564868	17.53339
ARIMA‐ETS‐THETA	5.339135	6.8117	0.06524217	19.59858
ARIMA‐ETS‐NNAR	5.831067	7.60046	0.07257552	21.47433
ARIMA‐ETS‐TBATS	4.723687	5.759178	0.05534035	17.26532
ARIMA‐THETA‐NNAR	5.776693	7.53166	0.0w7194921	21.27545
ARIMA‐TBATS‐NNAR	5.196069	6.553253	0.06281116	19.04652

Table 7 indicates the Model Confidence Set (MCS) procedure, calculated at a 95% confidence level (*α* = 0.05 *α* = 0.05), identified a Superior Set of Models (SSM) consisting of eight candidates that demonstrated statistically indistinguishable forecasting accuracy. The ARIMA‐ETS‐TBATS hybrid ensemble was identified as the top‐ranked model (RankM = 1*RankM* = 1, pMCS = 1.000*pMCS* = 1.000), followed closely by the ARIMA‐ETS hybrid and the standalone ETS model, which also achieved perfect *p*‐values of 1.000. Notably, while the single ETS model exhibited the lowest absolute loss statistic (0.003), the MCS algorithm prioritized the ARIMA‐ETS‐TBATS ensemble as the most robust candidate overall. The remaining five models in the superior set, including standalone ARIMA and TBATS, displayed varying degrees of accuracy but remained statistically comparable to the top performers (*p* > 0.05; *p* > 0.05). In contrast, the standalone NNAR and Theta models, along with several of their specific hybrid combinations (such as ARIMA‐Theta‐NNAR), were eliminated from the set (*p* < 0.05; *p* < 0.05), providing sufficient statistical evidence that they yield significantly inferior predictive performance compared to the models retained in the Superior Set.

**Table 7:** Model confidence set (MCS) results.

Model	RankM	MCS p‐value	Loss statistic	Status
ARIMA‐ETS‐TBATS	1	1.000	0.613	Superior set
ARIMA‐ETS	2	1.000	0.505	Superior set
ETS	3	1.000	0.003	Superior set
TBATS	4	0.780	0.829	Superior set
ARIMA‐ETS‐NNAR	5	0.670	0.744	Superior set
ARIMA‐TBATS	6	0.338	0.919	Superior set
ARIMA	7	0.243	1.008	Superior set
ARIMA‐ETS‐Theta	8	0.213	0.958	Superior set
ARIMA‐TBATS‐NNAR	—	< 0.05	—	Eliminated
ARIMA‐NNAR	—	< 0.05	—	Eliminated
Theta	—	< 0.05	—	Eliminated
NNAR	—	< 0.05	—	Eliminated
ARIMA‐ Theta‐ NNAR		< 0.05	—	Eliminated
ARIMA‐ Theta		< 0.05	—	Eliminated

As shown in Table 8 The Diebold–Mariano (DM) test was conducted to assess whether the forecasting accuracy of the ARIMA and ARIMA–ETS–TBATS models differed significantly from that of the TBATS benchmark. The TBATS model served as the reference, yielding a DM statistic of 0.000 (*p* = 1.000), consistent with no difference when compared to itself. In contrast, the ARIMA model produced a significantly negative DM statistic (DM = –4.315, *p* = 0.0035), indicating that ARIMA achieved significantly lower forecast errors than TBATS at the 1% significance level. Similarly, the ARIMA–ETS–TBATS hybrid showed an even stronger improvement in predictive accuracy (DM = –5.663, *p* = 0.0008), also statistically significant at the 1% level. These results indicate that both ARIMA and the ARIMA–ETS–TBATS hybrid significantly outperform TBATS in terms of predictive accuracy, with the hybrid model achieving the greatest reduction in forecast error among the compared models. DM testing showed that both ARIMA (DM = –4.315, *p* = 0.0035) and the ARIMA–ETS–TBATS hybrid (DM = –5.663, *p* = 0.0008) significantly outperformed the TBATS benchmark in forecasting accuracy. The hybrid model exhibited the largest improvement.

**Table 8:** Diebold–Mariano test for equal predictive accuracy.

Models	DM statistics	*p* value
TBATS	0.000	1.0000
ARIMA	−4.315	0.0035
ARIMA‐ETS‐TBATS	−5.663	0.0008

Table 9 displays the forecasted incidence of tuberculosis (TB) per 100,000 population in Somalia from 2023 to 2030, modeled using the ARIMA‐ETS‐TBATS hybrid approach. The point forecasts exhibit a gradual decline in TB cases, starting at approximately 242.1 in 2023 and decreasing to about 214.9 by 2030. The 80% and 95% confidence intervals illustrate the ranges of uncertainty surrounding these projections, with the 95% lower and upper bounds indicating that TB incidence could fluctuate between approximately 183.6 and 247.2 cases per 100,000 by 2030.

**Table 9:** Forecast Tuberculosis incidence per 100,000 population during 2023 to 2030 using the best‐fitted model of HYBRID ARIMA‐ETS‐TBATS.

Model	Year	Point forecast	80% lower	Higher 85%	95% lower	Upper 95%
ARIMA‐ ETS‐ TBATS	2023	242.0743	238.7934	245.4518	237.0310	247.2142
ARIMA‐ ETS‐ TBATS	2024	238.1489	232.7745	243.7160	229.8784	246.6121
ARIMA‐ ETS‐ TBATS	2025	234.2364	226.7138	242.0220	222.6619	246.0738
ARIMA‐ ETS‐ TBATS	2026	230.3366	220.5356	240.4454	215.2658	245.7152
ARIMA‐ ETS‐ TBATS	2027	226.4496	214.2228	239.0035	207.6637	245.5625
ARIMA‐ ETS‐ TBATS	2028	222.5752	207.7726	237.6989	199.8516	245.6200
ARIMA‐ ETS‐ TBATS	2029	218.7135	201.1871	236.5297	191.8325	245.8843
ARIMA‐ ETS‐ TBATS	2030	214.8643	194.4699	235.4921	183.6120	246.3500

Table 10 presents the projected incidence of tuberculosis (TB) per 100,000 population in Somalia from 2023 to 2030, utilizing the TBATS model. The point forecasts indicate a gradual decrease in TB incidence, starting from approximately 242.1 cases in 2023 and declining to about 215.6 cases by 2030. The accompanying confidence intervals, with 80% and 95% levels, suggest varying ranges of uncertainty around these estimates, with the 95% upper and lower bounds providing insights into the potential fluctuations in TB incidence. For instance, by 2023, the incidence could realistically range from about 237.8 to 246.4 cases per 100,000, with the trend indicating a continued reduction over the following years

**Table 10:** Forecast Tuberculosis incidence per 100,000 population during 2023 to 2031 using the best‐fitted model (TBATS).

Model	Year	Point forecast	80% lower	Higher 85%	95% lower	Upper 95%
TBATS	2023	242.1005	239.3160	244.9047	237.8499	246.3971
TBATS	2024	238.2052	234.1913	242.2608	232.0833	244.4246
TBATS	2025	234.3483	228.7772	240.0013	225.8610	243.0271
TBATS	2026	230.5297	223.1770	238.0282	219.3433	242.0569
TBATS	2027	226.7493	217.4482	236.2886	212.6203	241.4357
TBATS	2028	223.0068	211.6276	234.7511	205.7501	241.1173
TBATS	2029	219.3022	205.7409	233.3944	198.7745	241.0717
TBATS	2030	215.6353	199.8086	232.2029	191.7262	241.2773

**Figure 7:**
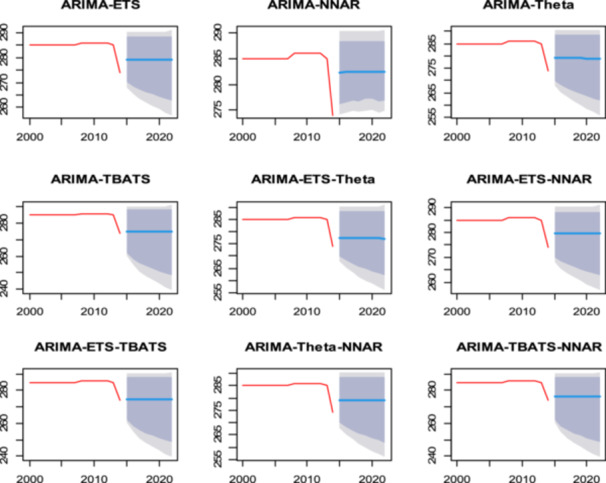
Forecast Tuberculosis incidence per 100,000 population (blue line) from ARIMA‐ ETS, ARIMA‐NNAR, ARIMA‐ Theta, ARIMA‐ TBATS, ARIMA‐ ETS‐ Theta, ARIMA‐ ETS‐NNAR, ARIMA‐ ETS‐ TBATS, ARIMA‐ Theta‐ NNAR, ARIMA‐TBATS‐NNAR.

As shown Figure 7, the forecasted tuberculosis (TB) incidence per 100,000 population from 2000 to 2020, depicted through various ARIMA modeling approaches, reveals significant differences in projected trends. The ARIMA‐ETS and ARIMA‐NNAR models indicate a gradual decline in TB incidence, suggesting effective public health interventions, while models such as ARIMA‐TBATS present a more stable forecast with slight fluctuations, indicating persistent TB challenges. The ARIMA‐ETS‐Theta and ARIMA‐ETS‐NNAR models exhibit similar trends, suggesting a cautious optimism. However, ARIMA‐Theta and ARIMA‐TBATS‐NNAR appear less optimistic, predicting higher incidence rates.

**Figure 8:**
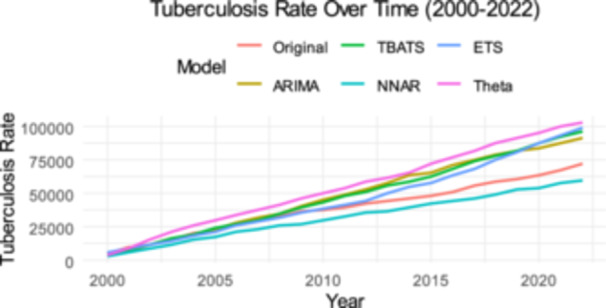
Forecast Tuberculosis incidence per 100,000 population from ARIMA, ETS, Theta, BATS, NNAR.

Figure 8 illustrates the tuberculosis rate over time from 2000 to 2022, comparing the original data with several forecasting models: TBATS, ETS, ARIMA, NNAR, and Theta. The original data, represented by the pink line, shows a consistent upward trend in tuberculosis rates, suggesting a growing public health concern. Each forecasting model captures this trend with varying degrees of accuracy. The ARIMA (yellow) and NNAR (blue) models closely follow the original data, indicating their effectiveness in capturing underlying patterns. The TBATS (green) model, while also showing an upward trajectory, slightly diverges from the original data, suggesting potential limitations in its fit. The Theta model (cyan) appears to predict a more gradual increase, possibly underestimating the actual rise.

**Figure 9:**
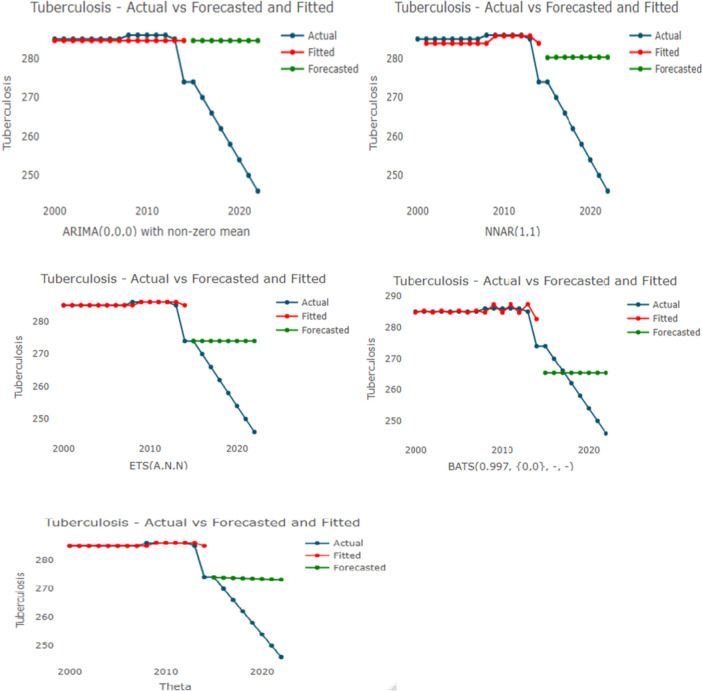
Actual, fitted, and forecasted values from ARIMA, TBATS, Theta‐NNAR, ETS, models the *x*‐axis and *y*‐axis are the year and tuberculosis incidence per 100,000 population.

As shown in Figure 9, the actual tuberculosis incidence data is plotted against the fitted and forecasted values generated by various models—ARIMA, TBATS, Theta, NNAR, and ETS. *A comparative* evaluation of the plots provides a definitive judgment on the relative performance of these modeling techniques.

The TBATS model emerged as the best‐performing model among those illustrated. It demonstrates the most robust alignment between the fitted values (red line) and the actual historical data (blue line), effectively capturing the complex underlying patterns and the recent shift in the incidence trajectory. In contrast, the ARIMA, NNAR, and ETS models performed poorly. These models failed to detect the changing dynamics of the time series, producing static, flat‐line forecasts that do not account for the observed downward momentum. Therefore, while the other models suggest an unrealistic stagnation, TBATS provides a superior and more adaptive fit for the dataset.

**Figure 10:**
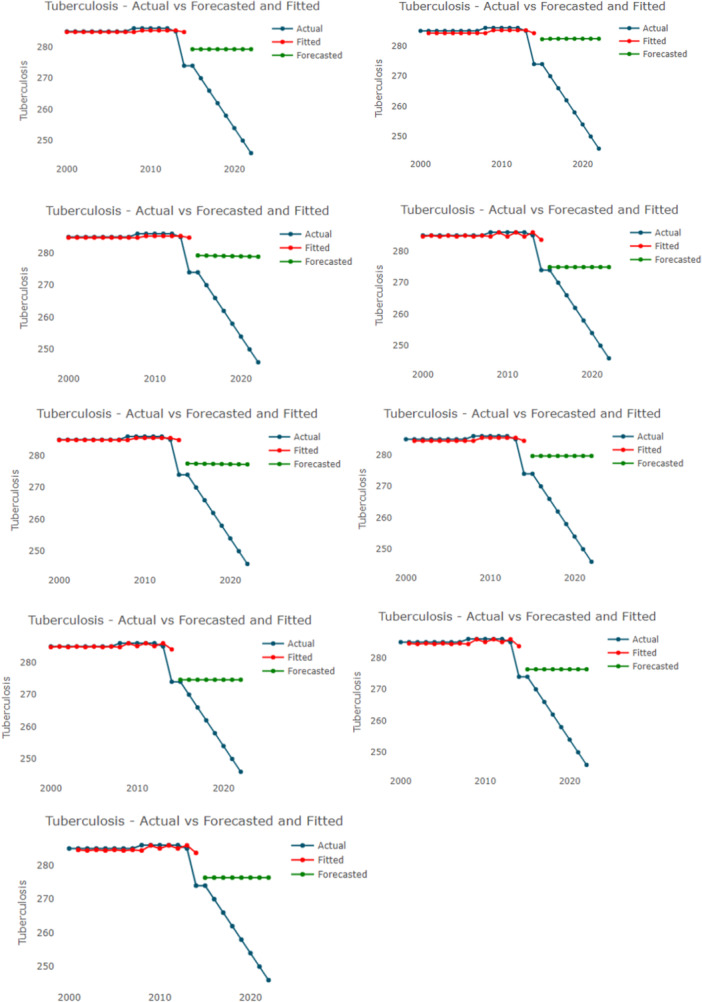
Actual, fitted, and forecasted values from ARIMA‐ETS, ARIMA‐NNAR, ARIMA‐ Theta, ARIMA‐ TBATS, ARIMA‐ ETS‐ Theta, ARIMA‐ETS‐ NNAR, ARIMA‐ ETS‐ TBATs, ARIMA‐ Thea‐ NNAR, ARIMA‐ TBATS – NNAR models the *x*‐axis and *y*‐axis are the year and Tuberculosis incidence per 100,000 population.

Figure 10 showcases the actual, fitted, and forecasted Tuberculosis incidence rates per 100,000 population over the years using a variety of predictive models. To facilitate specific model evaluation, each panel is distinct, representing a specific hybrid configuration such as ARIMA‐ETS, ARIMA‐NNAR, or ARIMA‐TBATS. The x‐axis represents the years, while the y‐axis indicates the incidence rates, allowing for a visual assessment of how closely the fitted values align with the actual data.

A definitive judgment on the relative performance of these ensembles identifies the Hybrid ARIMA‐ETS as the best‐performing model. Among all combinations tested, the ARIMA‐ETS framework demonstrated the superior ability to capture the complex structure of the time series, providing the most accurate alignment with historical trends. In contrast, the ARIMA‐NNAR model performed poorly. This specific hybrid combination failed to adequately replicate the underlying epidemiological dynamics, resulting in a less robust fit compared to the optimal ARIMA‐ETS ensemble.

## 4 Discussion

In this study, we evaluated 14 forecasting models for TB incidence in Somalia using annual data from 2000 to 2022, including five single models (ARIMA, ETS, TBATS, Theta, NNAR) and nine hybrid ensembles. Among the single approaches, TBATS yielded the strongest performance, while the ARIMA–ETS–TBATS hybrid consistently produced the most accurate and stable forecasts across schemes. The superior performance of TBATS and its hybrid variants suggests that Somalia's TB incidence data exhibit irregular seasonality, structural variability, and nonlinear trends that are not well captured by conventional linear models. These patterns likely reflect real‐world contextual challenges in Somalia, including weak surveillance systems, conflict‐related disruptions, and inconsistent reporting. Deterministic accuracy metrics such as Willmott's Index, percentage bias, and explained variance also confirmed the reliable agreement between predicted and observed values. Similarly, a previous study employed this to measure agreement predicted and observed models performance [30]. Moreover, another study used deterministic performance to measure the agreement between predicted and observed values [31]. Both the Hybrid and TBATS models project a gradual decline in TB incidence, decreasing from approximately 242 cases per 100,000 in 2023 to roughly 215 by 2030. However, this slow reduction trajectory indicates that Somalia is currently off‐track to meet the aggressive reductions required by the WHO End TB milestones for 2025, 2027, and 2030. Consequently, the disparity between this forecasted stabilization and the global elimination targets underscores an urgent need for intensified public health interventions to accelerate the rate of decline.

Our findings contrast with studies in regions like Malaysia and India, where more conventional ARIMA and NNAR models were identified as most suitable [32]. This divergence likely highlights differences in the underlying data structure; while TB incidence in more stable settings may follow predictable patterns amenable to standard models, the outperformance of the TBATS model in Somalia suggests a more complex time series. The TBATS model is specifically designed to handle features like multiple or non‐integer seasonality and significant data noise, which are plausible characteristics of incidence data from a region with long‐term instability and reporting inconsistencies. Therefore, its success indicates that Somalia's data contains irregular fluctuations that are not well‐captured by the more rigid assumptions of models that performed best elsewhere.

The projected downward trajectory in TB incidence is a statistical extrapolation from historical data and does not explicitly account for external epidemiological drivers such as changes in diagnostics, treatment adherence programs, or operational capacity of the health system. Therefore, this forecast should be interpreted conditionally: it assumes that past structural factors influencing TB reduction will remain constant. Any disruption in health services, conflict escalation, population mobility, or reporting instability could cause significant deviations from the expected trend, especially in fragile public health environments like Somalia. Recent studies have highlighted the complexity of tuberculosis dynamics in low‐resource and conflict‐affected settings, where surveillance quality, population displacement, and socioeconomic conditions create substantial variability in incidence patterns [11]. Hybrid and advanced ensemble approaches have shown improved accuracy for TB forecasting in such environments because they better capture irregular trends and non‐stationary fluctuations, supporting the suitability of our ARIMA–ETS–TBATS ensemble for the Somali context. Another study in Ethiopia revealed the ARIMA (2,2,0) model as the most suitable for forecasting tuberculosis incidence in Tigray, achieving a root mean squared error (RMSE) of 0.08 and demonstrating its effectiveness for future predictions with a Box‐Ljung test *p*‐value of 0.8668, indicating no significant lagged autocorrelations in the residuals. Study in Kenya reveal ARIMA‐ANN hybrid model demonstrated significantly better predictive and forecast accuracy compared to the ARIMA model for childhood TB incidence [1].

Study in Nigeria found The ARIMA model identified for predicting under‐5 mortality rates in Nigeria was ARIMA (0, 3, 2), which was selected based on its lowest Akaike's Information Criteria (AIC) and Bayesian Information Criteria (BIC) values, indicating its appropriateness for the data, and it demonstrated strong predictive performance with a root mean squared error (RMSE) of 0.23 and a modified Nash‐Sutcliffe efficiency coefficient of 0.996, reflecting its accuracy in capturing the underlying trends in the historical mortality data. The study in Taiwan found that the SARIMA‐ETS hybrid model demonstrated superior short‐term forecasting accuracy for TB incidence rates (RMSE: 0.084%, MAE: 0.067%, MAPE: 0.646%, MASE: 0.870%), while the ETS model projected the most significant long‐term reduction in TB epidemics by 2025 and 2030.

Study in Ghana The study focused on estimating the incidence of tuberculosis (TB) cases at the Korle‐Bu Teaching Hospital in Ghana, utilizing a time series analysis with the Box‐Jenkins approach, which identified the ARIMA (1, 0, 1) model as the best fit for the data, based on the lowest Akaike Information Criterion (AIC) and Bayesian Information Criterion (BIC) values, indicating that the model effectively captured the underlying patterns in the monthly TB cases reported from 2008 to 2017, despite the absence of a clear increasing or decreasing trend in the data. The study analyzed tuberculosis incidence in Anhui province using the ARIMA (0, 1, 1) (0, 1, 1) 12 model, which effectively captured seasonal and non‐seasonal trends, revealing that the highest reported TB rates occurred in March, with significant variations across regions, particularly in Tongling, Chizhou, and Huainan, where the incidence rates were notably high [2]. The forecasted decline is a purely statistical extrapolation of historical data and is not explicitly linked to real‐world interventions like WHO programs or improved diagnostics. Our model implicitly assumes that the unspecified positive drivers responsible for past reductions will continue, an assumption that is critical to acknowledge. Therefore, the plausibility of this downward trend is highly dependent on the sustained capacity and stability of Somalia's fragile healthcare system, which remains a significant uncertainty.

### 4.1 Study Limitations

A key limitation of this study is the exclusive use of annual univariate tuberculosis (TB) incidence data, resulting in a relatively small sample of 23 observations (2000–2022). Although this format was determined by data availability in Somalia, it restricts the analytical flexibility of the models and increases the risk of overfitting, especially for neural‐network‐based models such as NNAR. The predictive capacity of the hybrid ensembles must therefore be interpreted cautiously in light of this limited sample size.

The absence of exogenous epidemiological predictors—such as HIV co‐infection, malnutrition, poverty indicators, healthcare access, and conflict intensity—also restricts the public health interpretability of the forecasts. Relying solely on historical TB trends omits important structural drivers of transmission dynamics, especially in fragile and conflict‐affected environments such as Somalia. As such, future research should aim to incorporate multivariate time‐series frameworks once reliable covariates become available.

In Somalia, TB surveillance systems face chronic underreporting, fragmented institutional capacity, population displacement, and intermittent data collection. These conditions introduce uncertainty and potential instability into reported incidence figures, which the statistical models cannot fully correct. Moreover, structural breaks due to conflict, famine, epidemics, and policy disruptions are not explicitly modeled in the current framework; thus, the confidence intervals presented here reflect statistical uncertainty but do not capture the broader systemic uncertainty associated with real‐world instability.

From a methodological perspective, the study employed classical statistical models and standard hybrid ensembles. More advanced methodologies—such as long short‐term memory networks (LSTM), attention transformers, or residual‐driven hybrid learning—were not applied due to the small sample size and lack of higher‐frequency surveillance data. These contemporary algorithms typically require hundreds of observations to avoid parameter instability and overfitting, making them unsuitable for the present dataset. As surveillance improves and monthly or weekly TB notifications become available, future research should prioritize the application of such advanced machine‐learning and deep‐learning architectures.

Additionally, rolling‐origin or blocked cross‐validation techniques could not be systematically implemented given the limited number of observations, restricting the full evaluation of temporal generalizability. Therefore, although hybrid approaches improved forecast robustness under constrained conditions, the long‐term projections must be interpreted within the context of limited data granularity, health‐system instability, and evolving public health conditions.

## 5 Conclusion

Employing a comprehensive suite of time series models has yielded actionable insights for forecasting tuberculosis in Somalia. The ARIMA‐ETS‐TBATS hybrid model emerged as the most effective tool, adeptly capturing the complex seasonal patterns within the incidence data. Crucially, these forecasts provide a direct guide for public health interventions; for example, officials can use the identified seasonal peaks to proactively time awareness campaigns and pre‐position resources for maximum impact. This study thus underscores how advanced modeling can transform historical data into a strategic tool, enabling a shift from reactive monitoring to proactive, evidence‐based control of tuberculosis in Somalia. Given that Somalia's tuberculosis epidemic is driven by a complex interplay of factors, future research should focus on enhancing predictive models. We propose the integration of key covariates, such as HIV prevalence and malnutrition rates, into hybrid modeling frameworks to better capture these dynamics. This approach would significantly improve model accuracy, leading to more robust projections and better‐informed public health strategies.

## 6 Policy Implication and Recommendations

The forecasting results generated by this study have important implications for tuberculosis (TB) control and public health planning in Somalia. First, the projected incidence trajectory provides health authorities with a forward‐looking evidence base that can improve preparedness, budget forecasting, and allocation of diagnostic supplies. Rather than reacting to case increases once they occur, health planners can anticipate demand and schedule procurement cycles accordingly.

Second, the forecasts should be incorporated into an early warning surveillance framework. If real‐time notification data begin to deviate persistently above the upper confidence limits of the projected values, this should be interpreted as a potential outbreak signal or service disruption. Such deviation could trigger rapid investigation, intensified screening, targeted community outreach, or operational audits to identify failures in treatment adherence or diagnostic access.

Third, although this study relied on annual data, the observed predictive patterns justify strengthening TB surveillance systems so that higher‐frequency data—monthly or weekly—can be captured. This would enable more responsive epidemiological forecasting, improved detection of seasonal peaks, and more precise targeting of interventions. Expanding electronic case reporting, integrating mobile health tools, and improving laboratory connectivity would greatly enhance the reliability and granularity of future TB forecasts.

Finally, the model outputs highlight the importance of addressing structural determinants of TB burden. As Somalia's epidemiological context remains shaped by conflict, displacement, poverty, and variable access to healthcare, policy planning should be coupled with investments in nutrition programs, housing stability, and continuity of treatment services for displaced populations. Strengthened surveillance and community‐level diagnostics should accompany any long‐term TB control policy. The study reveals that the projected rate of incidence reduction is critically insufficient to align with the ambitious milestones set by the WHO End TB Strategy for 2025 and 2030. Consequently, national policymakers must urgently pivot from maintaining current control measures to implementing aggressive, targeted interventions that significantly accelerate the decline to bridge this widening gap.

## 7 Recommendations

Based on our findings, to effectively combat tuberculosis (TB) in Somalia, we offer the following recommendations, emphasizing a data‐driven and adaptable approach to future control efforts. We must bolster existing public health initiatives by ensuring consistent implementation and rigorous monitoring, as our projections highlight that continued efforts are crucial for sustaining the decline in TB incidence. We should also invest in developing and maintaining a robust surveillance system to provide accurate and timely data collection. This system should be capable of capturing the complexities of TB transmission, including factors related to social determinants of health and access to healthcare. Furthermore, we need to implement a flexible and adaptive strategy that responds effectively to unexpected fluctuations in TB incidence, considering factors such as conflict, climate change, and drug resistance. Finally, we must strengthen collaboration between national and international stakeholders to improve program implementation, data sharing, and resource allocation.

## Author Contributions

**Farduus Ibraahim Mohamed:** investigation. **Saralees Nadarajah:** investigation, writing – original draft, writing – review and editing.

## Funding

The authors received no specific funding for this work.

## Consent

The authors have nothing to report.

## Conflicts of Interest

The authors declare no conflicts of interest.

## Transparency Statement

The lead author Saralees Nadarajah affirms that this manuscript is an honest, accurate, and transparent account of the study being reported; that no important aspects of the study have been omitted; and that any discrepancies from the study as planned (and, if relevant, registered) have been explained.

## Data Availability

The code can be obtained from the corresponding author. The data are publicly available at https://data.worldbank.org/indicator/SH.TBS.INCD.
